# Design and Analysis of the Domestic Micro-Cogeneration Potential for an ORC System Adapted to a Solar Domestic Hot Water System

**DOI:** 10.3390/e21090911

**Published:** 2019-09-19

**Authors:** Daniel Leal-Chavez, Ricardo Beltran-Chacon, Paola Cardenas-Terrazas, Saúl Islas, Nicolás Velázquez

**Affiliations:** 1 Centro de Investigación en Materiales Avanzados, S.C., CIMAV, Chihuahua 31136, Mexico; daniel.leal@cimav.edu.mx (D.L.-C.); paola.cardenas@cimav.edu.mx (P.C.-T.); 2 Instituto de ingeniería, Universidad Autónoma de Baja California, Mexicali 21100, Mexico; islass@uabc.edu.mx (S.I.); nicolas.velazquez@uabc.edu.mx (N.V.)

**Keywords:** solar domestic cogeneration, Organic Rankine Cycle, acetone, evacuated tube solar collector

## Abstract

This paper proposes the configuration of an Organic Rankine Cycle (ORC) coupled to a solar domestic hot water system (SDHWS) with the purpose of analyzing the cogeneration capacity of the system. A simulation of the SDHWS was conducted at different temperatures, observing its performance to determine the amounts of useable heat generated by the solar collector; thus, from an energy balance point of view, the amount of heat that may be used by the ORC could be determined. The working fluid that would be suitable for the temperatures and pressures in the system was selected. The best fluid for the given conditions of superheated vapor at 120 °C and 604 kPa and a condensation temperature of 60 °C and 115 kPa was acetone. The main parameters for the expander thermodynamic design that may be used by the ORC were obtained, with the possibility of generating 443 kWh of annual electric energy with 6.65% global efficiency of solar to electric power, or an overall efficiency of the cogeneration system of 56.35% with a solar collector of 2.84 m^2^.

## 1. Introduction

The growing demand for energy and the current dependence on fossil fuels require more efficient processes and technologies to be developed, to allow a transition into a system with sustainable energy sources. 

In general terms, the objective is that buildings have zero net energy consumption through an efficient use of energy, and the adoption of renewable sources of energy and other technologies [1]. Photovoltaic, solar, thermal, and biomass technologies can contribute to reducing consumption for heating of spaces in the Mediterranean region [2], in order to reduce high energy costs, create energy security, and mitigate local air pollution [3,4,5]. Solar energy systems allow consumption to be reduced to almost zero net energy in buildings. For combined heat and power (CHP) systems, return periods are between 5.5–6.5 years, while for photovoltaic systems it takes 7 years [6].

Organic Rankine Cycle technology has intensified in recent years, and is being used as the main alternative to convert energy at low temperatures [7].

Solar water heaters generally capture a greater amount of solar energy during seasons with low hot water consumption, such as summer. In these circumstances, a lot of solar energy is wasted.

Extending the utilization of energy captured by solar collectors through a second application would favor a technological alternative for electric production through renewable energies and a reduction of CO_2_ emissions associated with conventional fossil fuel-based generation techniques.

An option to improve the utilization factor of solar systems is by integrating ORC systems to generate electricity in standard domestic use. The ORC is a proven technique to convert unused low temperature heat into useable mechanical or electric energy [8], and is characterized by its simplicity and flexibility at a relatively low operating temperature [9]. 

This technique has been widely used in recent years with different heat sources, such as geothermal, heat recovery, biomass, and solar systems. There are many applications and configurations at different temperatures and capacities [10]. In recent years, there have been many studies on ORC, ranging from optimization, through component and working fluid selection, to techno-economic and experimental studies [11].

**Figure 1:**
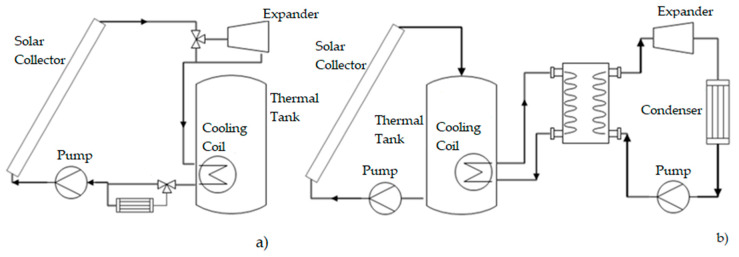
(**a**) Proposed configuration, (**b**) typical configuration.

Currently, there are a number of domestic-sized solar ORC systems [12,13,14,15,16]; their main operation characteristics are shown in Table 1. However, none of them has a configuration using direct vapor generation with evacuated tube collectors. These configurations generally use an indirect heat exchanger between the solar heating system and the ORC system (see Figure 1). The aim is to use the solar system only for pre-heating the working fluid, and to later evaporate the fluid with a conventional heat source under controlled conditions.

There are different studies in the field of optimization of residential solar ORC configurations. A system with flat plate collectors with a heat source of 3.542 kW [12] uses a heat exchanger for condensation and a subsystem for waste heating to heat the water in a hot water tank. A configuration of 100 m^2^ of a parabolic channel with ORC [13] uses a heat exchanger to transfer heat from the parabolic channel, and operates as an evaporator for the ORC. A configuration of an evaporator SDHWS used as an intermediate heat exchanger to heat the working fluid of the ORC system [14] additionally uses a system of indirect heating for the DHWS and three flat plate solar thermal collectors. A configuration with a solar area of 15 m^2^ and thermal energy storage [15] maintains and uses energy to mitigate solar energy variations in the ORC. A solar system with a linear Fresnel collector, with a total surface area of 148 m^2^, thermal storage with phase change, and an ORC [16], uses the tank indirectly for phase change as the evaporator from the ORC. 

The proposed system presents a fundamental difference to other applications reported in the literature, in that the heat transfer is carried out directly from the evacuated tube solar collectors, using it as an evaporator. This improves the cycle efficiency due to the elimination of heat losses in the intermediate heat exchanger. When intermediate heat exchangers are added, each heat exchanger requires a temperature difference to be effective. Therefore, each added heat exchanger decreases the maximum source temperature. In other words, the heat transfer increases the generation of entropy of the system, thus reducing the ability to produce work.

This configuration is simpler because it eliminates the need for using two separate systems (a solar system and ORC system), thus avoiding the need for two pumps to recirculate the fluid in both systems.

The objective is that the proposed system can simultaneously provide two services: hot water and electricity production, instead of using the solar collector to provide only one service (hot water). It also increases the annual thermal performance of the collector because of the utilization of extra heat for electricity production. The integration of an ORC system into a solar domestic hot water system (SDHWS) is presented to achieve a domestic micro-cogeneration, taking into consideration the pressures and temperatures at which these two systems may work properly. The energy of the fluid that does not produce work in the expander is utilized in the hot water tank for simultaneous production of hot water and electricity. 

**Figure 2:**
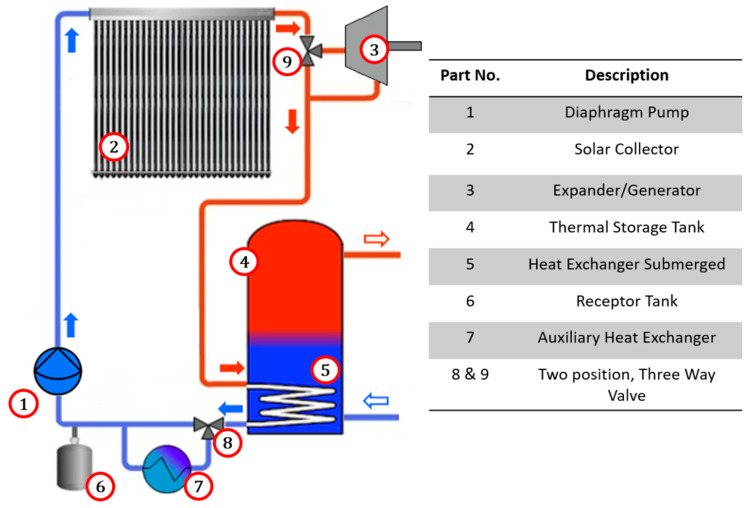
Scheme of ORC adapted to solar domestic hot water system (SDHWS).

The use of a variable flow pump is proposed for the vapor generation to occur directly in the solar collector, ensuring a constant vapor pressure and temperature at the collector outlet, while for the condensation zone of the system, a heat exchanger integrated within the SDHWS hot water tank will be used (see Figure 2).

There are a number of studies related to working fluid selection for the ORC [17,18,19,20,21]; however, they generally focus on operation temperatures. In contrast, in this study the (relatively low) operation pressures that the SDHWS may withstand were first considered, and later their influence on the fluid properties and the thermal performance system were evaluated. 

For the basic engineering design of this system a dimensioning method is proposed, including (A) an analysis of the thermal energy potential available in the SDHWS for a secondary use (ORC); (B) identification of operation conditions (the most adequate temperature and mass flow for an efficient operation of the SDHWS and ORC integrated system); and (C) the identification of working fluids that may allow a better performance to be obtained under these conditions.

For this proposal a year-long simulation was conducted in Transient System Simulation Tool (TRNSYS) of a conventional solar hot water system with an evacuated tube collector. The results of the simulation of the main components in the system were validated with experimental data available in the literature. In this analysis the year-long climatic characteristics, the hourly solar energy utilization in the collector, and the hot water needs for an average-sized household were considered. 

The results of the simulation in TRNSYS show the energy flows and their destination, classifying them into thermal losses, useable heat for hot water, and potential useable (unutilized) heat, as well as the amount of energy that the cogeneration system can produce is calculated. 

From this study, it is possible to analyze the system performance when working at different temperature levels and to estimate the cogeneration potential.

## 2. Description of the System

A cogeneration system is proposed for integration into solar water heating systems [22], as shown in Figure 2 and Table 2 describes in detail the main system components. It comprises a solar collector (evaporator) (2) which is interconnected to a two-position, three-way valve (9). In one position, the valve (9) allows the working fluid to flow towards the condenser (5), and in the other valve position the working fluid is directed towards an expansion device (3). The working fluid that comes out of the expansion device (3) is directed towards the condenser (5). The condenser (5) is a heat exchanger submerged inside the hot water tank (4).

The system uses an auxiliary heat exchanger (7), in which a two-position, three-way valve (8) directs the working fluid towards the heat exchanger when heat removal in the condenser (5) is not sufficient. When the system changes from heating mode to ORC mode, the fluid pump (1) reduces the flow, causing part of the working fluid to be stored in a receptor tank (6). 

One characteristic of the proposed system is that it may work under two operation modes; either water heating only, or combined heat and power production, depending on the requirements. 

### 2.1. Operation Mode: Water Heating

The system configuration for exclusive production of hot water requires that the working fluid at the collector output is directed towards the heat exchanger inside the hot water tank. Through the heat exchanger/condenser, the working fluid transfers heat indirectly (without direct contact) to the water in the hot water tank. To achieve an efficient operation, the working fluid mass flow is controlled so that the working fluid temperature at the collector output is higher than the preset average tank water temperature. The working fluid that leaves the condenser out of the hot water tank is then directed towards the fluid pump and then to the evaporator inlet. This cycle is operated as long as the working fluid temperature at the solar collector output is higher than the water temperature in the hot water tank, or until the preset temperature (60 °C) is reached. In this mode of operation, the expander is not used; the fluid instead flows directly from the collector to the submerged heat exchanger.

In this mode of operation, there is no phase change of the working fluid within the cycle, so the pump only circulates the working fluid, and thus it does not impose a high pressure change across the cycle.

### 2.2. Operation Mode: Organic Rankine Cycle 

The contribution of thermal energy to the water in the hot water tank when there is low hot water demand causes a progressive increase of water temperature in the hot water tank. Once the hot water tank temperature reaches a preset operation temperature (60 °C), the valve (9) (Figure 2) changes position and directs the working fluid towards the volumetric expander. Simultaneously, the pump turns off momentarily to drain the working fluid partially into the receptor tank (6), which stores the unused working fluid when working in the Rankine cycle. After this, the pump starts to operate with a mass flow that allows the production of superheated vapor (at 120 °C) at the evaporator outlet.

Variations in the solar resource and the useable heat produced require that the flow is regulated depending on the solar irradiance to produce steadily superheated vapor at the evaporator outlet. The expander may start working at a moment when there is a pressure differential between the hot side (collector outlet) and the cold side (hot water tank and/or condenser) of the system and convert some useful heat into useable mechanical work. This way, some of the solar energy captured by the solar collector which is not required to heat water (once it has reached a preset temperature) is used to produce mechanical work.

The working fluid at a high temperature at the expander output is used to heat the water in the hot water tank when the conditions of the hot water tank temperature allow it, thus achieving a cogeneration of electrical and thermal energy.

As heat is transferred into the hot water tank, its temperature increases, and the temperature difference between the working fluid and the hot water tank decreases; this eventually reduces the condenser cooling capacity to remove the necessary energy to condense the working fluid. Under these circumstances, it is necessary to have an auxiliary heat exchanger that allows the removal of the necessary energy to condensate the working fluid before it reaches the fluid pump. To achieve this energy removal, the valve (8) changes position so it directs the working fluid towards the auxiliary heat exchanger, where it is subcooled and stored in the receptor tank, completing the Rankine cycle. 

When all the energy provided by the collector is required for water heating, or when there is not enough irradiance for working fluid evaporation, the valve (9) changes position so the fluid flows directly towards the condenser in the hot water tank, thus homogenizing pressure in the whole system.

Heat transfer is conducted directly from the solar collector towards the ORC working fluid, and the fluid energy not extracted by the expander is conducted towards the hot water tank for the simultaneous production of hot water and electricity.

## 3. Thermodynamic Model of the SDHWS

**Figure 3:**
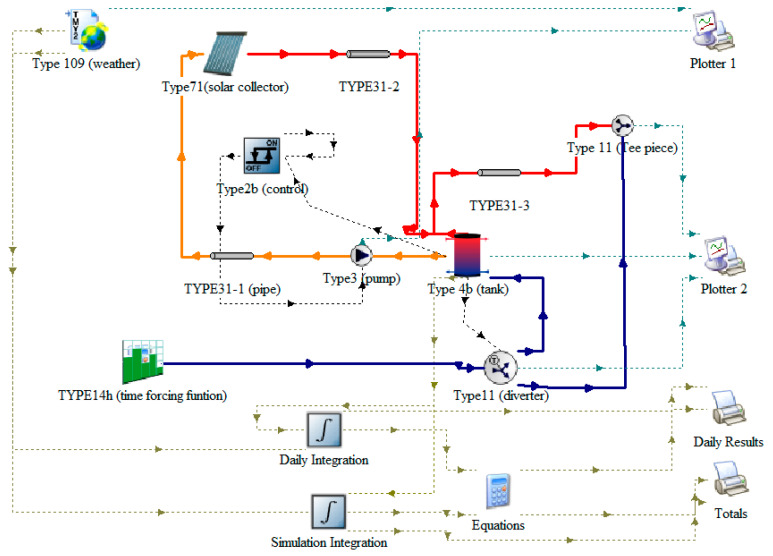
SDHWS scheme in the TRNSYS simulation studio.

The SDHWS was analyzed in TRNSYS, describing a conventional evacuated tube collector solar hot water system. These simulations allow the prediction of the performance of a system by analyzing performance under dynamic conditions, allowing a proper system sizing. Figure 3 shows the scheme used for the study of the TRNSYS simulation.

The weather data location used in this study was Chihuahua, Mexico, with 1/64 hour time intervals for the simulation in order to obtain consistent results.

The objective of this analysis was to characterize system performance at different operation temperatures, and to identify the parameters that influence heat gain. The model refers only to the operation of the SDHWS, without considering the operation within the ORC. The focus of this simulation is to analyze the collector useful energy and the domestic hot water energy requirements in order to estimate the energy that could be used within the ORC system.

The results allow for a quantification of the heat transfer rate, the extra heat that could be harnessed for a secondary use, and the mass flows and physical conditions of working fluids that will be used in the system.

The collector converts solar irradiance into useable heat and transfers it to the working fluid that flows through the collector manifold towards the cooling coil submerged in the hot water tank, where it heats water for domestic use. The pump keeps working until the top of the hot water tank reaches an operation temperature of ~75 °C, as recommended by the manufacturer [23]. If the hot water does not reach the desired temperature upon its utilization, it may be heated by an auxiliary conventional heater.

The operation and simulation conditions for the main components (i.e., solar collector, hot water tank, and internal heat exchanger) were validated with experimental data available in the literature [24,25,26]. The results shown, verify that the models have been correctly implemented in the simulation, and also show the detail of each one.

### 3.1. Solar Collector

Solar collectors are a special type of heat exchangers that transform solar radiation into useable heat, which is transferred into a fluid that circulates through the collector [27].

**Figure 4:**
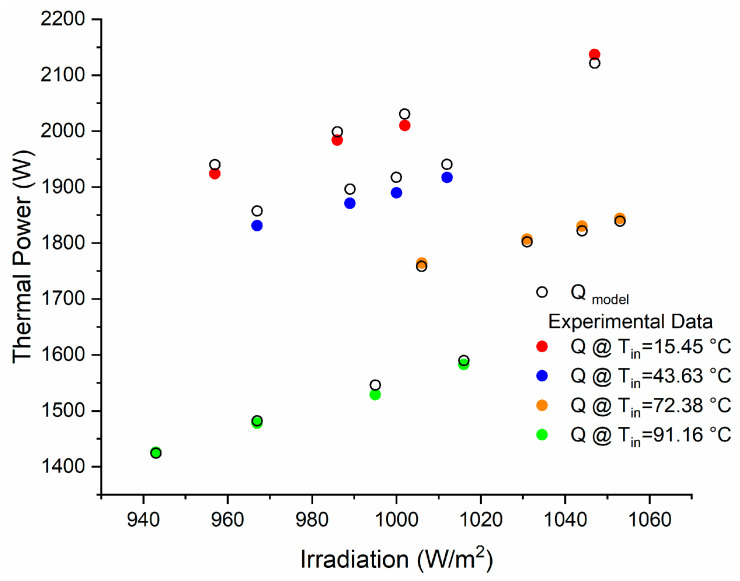
Comparison of experimental tests versus the numerical model of the solar collector.

The results obtained in the field tests conducted by Kottwitza [28] were considered for the validation of the mathematical model used in this study, while *Q_model_* shows the results obtained through the model used by type 71, as shown in Figure 4.

As may be seen in Figure 4, there are very close approximations to the values obtained in the collector experimental data, with a maximum error of 1.44% at the outlet power.

### 3.2. Hot Water Tank

The hot water tank is an important component of a solar hot water system since it may considerably improve its efficiency and profitability, allowing better use of the solar system, and equating the useful heat of the solar resource and energy demand [29]. Solar energy is a time-dependent energy resource, just like the energy requirement, but both occur at different times than the solar energy supply; therefore, energy storage is necessary if solar energy is to be used to meet a major part of energy needs.

**Figure 5:**
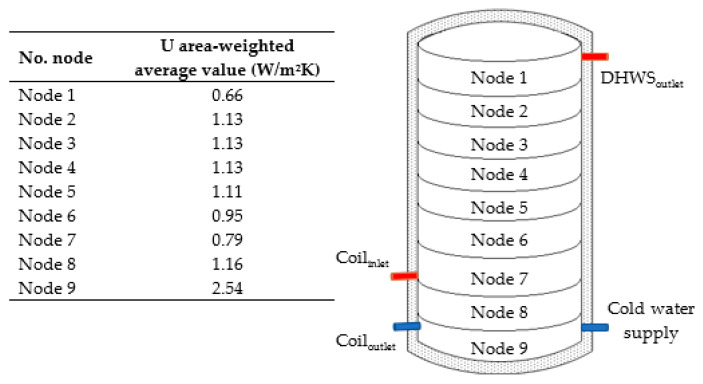
*U* coefficient weighing by nodes in the hot water tank.

For the validation of the hot water tank model and submerged cooling coil, the experimental data in the literature were compared to data obtained through the simulation. The results obtained by Cruickshank and Harrison [29], who used a commercial 270 L hot water tank, were considered. This tank uses fiberglass insulation with a 0.047 m thickness, and a 0.036 W/mK thermal conductivity. The hot water tank has a height of 1.5 m and a 0.45 m internal diameter. In the experiment, the water was heated and mixed at a temperature of 54.0 °C in an environment of 20 ± 0.03 °C over 48 h. Readings were carried out every 3 min, with temperature sensors spaced every 15 cm in a vertical array to obtain stratification readings. The heat loss coefficients for each node were considered for the validation of this model, as shown in Figure 5.

From the data of the experimental cool down test conducted by Cruickshank and Harrison [29], a loss of 14,580 kJ was obtained over 48 h. The result of the simulation of type 60 was 14,835 kJ, with an error of 1.74%, which shows that the mathematical model of the hot water tank is very accurate.

From the same loss calculation, taking a single average loss coefficient from the insulation characteristics, *U* = 0.86 W/m^2^K, a significantly reduced heat loss is obtained, with a 19% difference with respect to the experiment.

### 3.3. Cooling Coil

The hot water tank sub-system for water heating in this study consists of a cooling coil submerged in the storage tank, where the high temperature working fluid coming from the solar collector exchanges heat with the water in the hot water tank.

Data presented by R. Farrington [24] were used for the validation of the cooling coil. This cooling coil has a total length of 18.6 m and an area of 0.929 m^2^; it is made of a plain copper tube with an internal diameter of 1.27 cm and an external diameter of 1.59 cm. 

The experimental data presented by R. Farrington [24] were taken into account to validate the heat exchanger simulation. 

**Figure 6:**
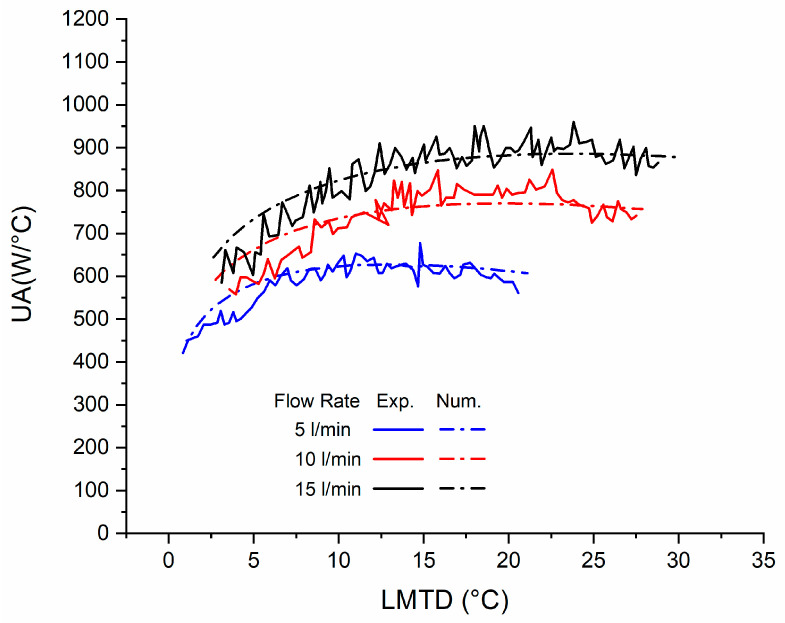
Comparison of experimental data versus TRNSYS of the submerged cooling coil.

The curves displayed by the model developed in TRNSYS by type 60 show a very good adjustment with respect to the experimental data obtained by R. Farrington [24], with an average difference of 1.6% between the three models (Figure 6).

## 4. Energy Balance

It is a common practice to use computer simulation to predict the performance of a solar domestic hot water system. This process is based on the precise specification of the system characteristics, with the purpose of integrating all these sub-systems and to analyze the behavior that would be obtained through the interaction of all these components in a time-variable transitory state, according to the weather changes at the site. 

### 4.1. Operation of the Solar SDHWS 

A typical meteorological year (TMY) of Chihuahua, Mexico, was considered for the simulation of the SDHWS; and the solar energy captured by the collector and the hot water needs were assumed for an average-sized household in Mexico. 

The required heat for a year-long supply of hot water was determined after analyzing the requirements, as well as the main devices in the system and the collector performance. In this study are considered the requirements of an average-sized household in Mexico of 3.7 (~4) members, according to INEGI [25]. A daily average hot water consumption of 236 L is assumed (Table 3), at an average temperature of 45 °C [26].

Considering Table 3, the hourly consumption is adjusted to the typical household consumption reported by ASHRAE [30]. A daily consumption of 236 L at 45 °C was considered, representing 86,140 L and 4464 kWh of thermal energy per year.

Three energy inputs were considered to obtain this required heat, in addition to the heat losses in the system: the solar collector, the auxiliary system, and the network water supply.

In the system, using type 31, a piping section of 10 m and 1.27 cm in diameter from the hot water tank to the tap was considered, located outdoors with a thermal insulation of 13 mm. For the connection between the solar collector and the hot water tank, 5 m of piping with a 19 mm thickness was used. For the thermal conductivity of the insulated copper tube, the data reported by Marini et al. [31] of *k* = 0.031 W/mK was used. The results obtained show values similar to those reported by ASHRAE [30] of *UA* = 0.346 W/mK, *U* = 8.48 W/m^2^K, based on the internal diameter of the pipe. 

The system must maintain the water in the hot water tank at a minimum temperature of 60 °C to avoid the growth of bacteria such as Legionella [26,30]. Therefore, there will be heat losses at the hot water tank which should be accounted for by the water heating system. These temperatures may vary depending on the internal temperature of the hot water tank and the ambient temperature.

**Figure 7:**
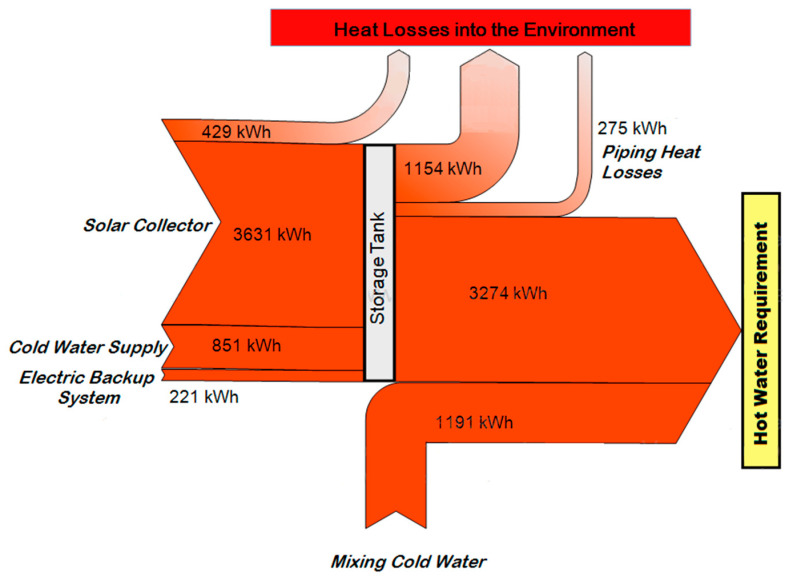
Energy balance in the domestic hot water system.

Taking these data into consideration, as well as the behavior of the types of components described above, the simulation was carried out in TRNSYS, obtaining the energy balance shown in Figure 7. The energy inputs for each inlet, as well as the heat losses for each part of the system, can be identified.

Under these conditions, the solar collector and the water supply input at ambient temperature provides 96% of the required energy, with a total energy of 5673 kWh, whereas the auxiliary heater must provide the remaining 4% with 221 kWh to meet the annual hot water requirement. There are partial losses through the environment of 1858 kWh, taking into consideration the losses of the solar collector, the hot water tank, and the supply pipes. 

However, the solar collector may supply a greater amount of energy if the re-circulation pump is kept operating during sunny hours; that is, not turning off the recirculation pump when the water reaches the set temperature point. This may be attractive if this useable heat is used in another process, such as an ORC.

### 4.2. Collector Performance at Different Temperatures

As the temperature difference between the working fluid and the ambient temperature increases, the collector efficiency decreases. However, there is a higher gain of enthalpy in the working fluid through a lower volumetric flow. 

Taking into consideration that one of the objectives of this study is to identify the thermal potential of a solar collector for a secondary use, additional to water heating, it is necessary to analyze the collector thermal performance at different mass flows and temperatures. Identifying these conditions will allow an estimation of the different working fluid performances under these circumstances. For cogeneration applications, these conditions involve superheated vapor at the evaporator (collector) and subcooled liquid at the condenser.

For a higher efficiency in the ORC, a higher pressure difference is required; therefore, the aim is to have the highest permissible pressure in the system. In the case of this study, a maximum permissible pressure of 800 kPa is considered in the collector, with a stagnation temperature of 228 °C according to the manufacturer data sheet [23]. In the condenser the objective is to condense with the hot water tank operation temperature at a relatively low pressure. This way, different scenarios may be analyzed considering different operation temperatures, in order to select the best operating condition.

The condensation temperature parameter has a direct impact on the system performance. Therefore, when defining the condensation temperature, it should be considered that setting a temperature lower than 60 °C implies that a certain amount of energy should be removed from the system through an external heat exchanger. These may compromise the amount of useable energy for heating water and increase the need for auxiliary heating.

On the other hand, if there is an exceedingly high temperature, the heat losses in the piping and hot water tank may increase, decreasing the collector efficiency. Thus, it is necessary to find a point where the need for the required useable energy may be met, and at the same time it operates at temperatures and pressures that allow efficient operation in an ORC.

When a constant temperature is required and operation is continuous in a transitory state, it is necessary to have a control system for regulation. In order to do this a variable flow pump can be considered, which is able to control the system optimally. The considered expander must work with a variable mass flow as well, where the expander would have a constant pressure and temperature conditions, and by having the expander of a fixed built-in volume ratio, the angular speed would increase or decrease depending on operating conditions.

For the Rankine cycle, it is desirable to obtain the lowest temperature cycle to keep the heat losses as low as possible, so the analysis is focused on inlet temperatures of 40, 50, and 60 °C. For the collector simulation, the input fluid was evaluated at different initial temperatures. A variable flow pump with a feedback controller was used to obtain a constant output temperature at different irradiances. 

**Figure 8:**
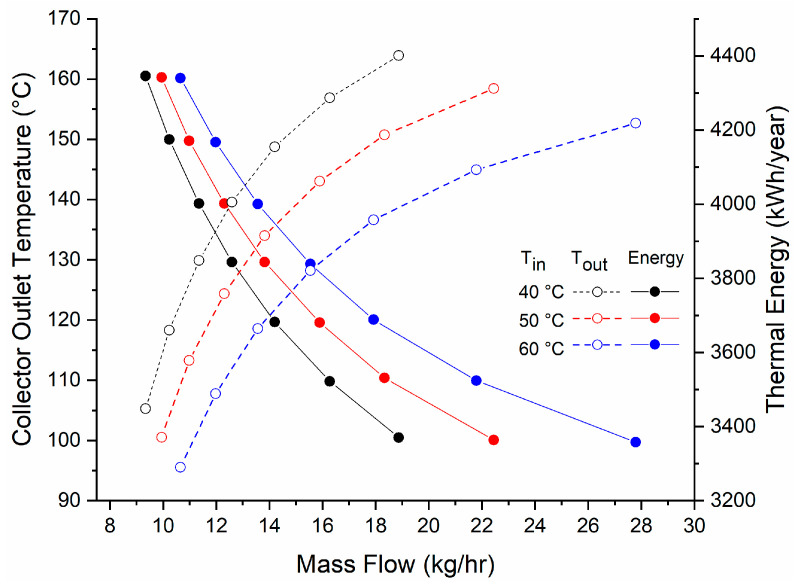
Energy produced at different operation temperatures.

Figure 8 shows the thermal energy produced annually with each established temperature difference, and the required mass flow.

## 5. ORC Efficiencies

To select the operation temperatures in the cycle, it is necessary to analyze the performance of the heat source (solar collector), as well as the ORC, when operating at such temperatures. A point of operation that allows the solar collector to have enough efficiency to supply the energy required by the SDHWS and for the ORC to have a good efficiency is desirable.

To condense at a temperature lower than 60 °C in the Rankine cycle, it is necessary to remove energy from the system, which implies a waste of the solar energy captured by the collector; therefore, the total system efficiency decreases. Condensing at temperatures ≥ 60 °C allows the utilization of a greater amount of heat for the generation of electric power, without compromising the energy required to keep the water at 60 °C.

**Figure 9:**
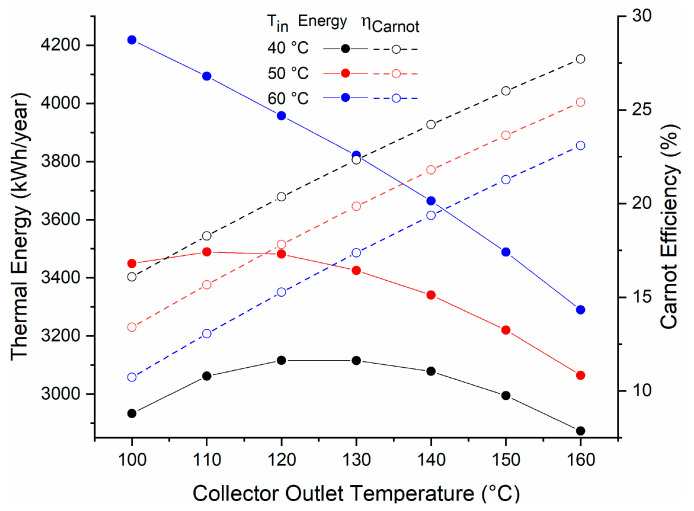
Energy and efficiency of the cycle at different operation temperatures.

With an input temperature of 60 °C and an output temperature of 120 °C, this allows us to obtain a useful energy of 3960 kWh and a Carnot efficiency of around 15% (Figure 9). 

Type 71 simulates solar collectors in which heating a working fluid occurs without phase change. Collector thermal efficiency and thermal losses depend mainly on the fluid average temperature. The energy balance in the solar collector when operating in ORC mode, where phase change occurs, takes into account the latent heat of vaporization. 

When analyzing solar collectors with phase change, the filling factor is considered. The filling factor is the ratio of volume occupied by liquid with respect to the total volume of the evaporator. A filling factor near to one indicates that most of the volume is occupied by liquid. A filling factor close to zero indicates that most of the volume is occupied by vapor, which can involve overheating of the fluid and increase the average temperature, affecting the calculation of thermal efficiency. 

An experimental study [26] conducted with a filling factor of 55% showed a decrease in the thermal efficiency of only 4.5%, compared to a collector without phase change. This shows that the thermal efficiency of the solar collector depends mainly on the average temperature, even when the heat transfer coefficients with phase change are very different.

In this study, the vapor superheated temperature (120 °C) is close to saturation temperature (119.6 °C). Therefore, it is considered that the variation of mean temperature over thermal efficiency is negligible. Thus, several authors have used different configurations to use collectors as evaporators, obtaining good efficiencies [32,33,34].

### 5.1. Working Fluid Selection

A great number of organic fluids with a boiling temperature lower than water may be used to operate with low temperatures and pressures in an ORC. A proper selection of the working fluid in an ORC is extremely important, since it will not only affect the cycle efficiency, but also the size of components, the expander design and the system stability. 

Firstly, for a proper selection of the working fluid, a practical pressure and temperature are defined within the cycle limits. A high pressure ratio favors cycle efficiency. In this study, the highest pressure is limited by the solar collector maximum operating pressure. It is important to also consider the isentropic efficiencies that would be obtained in the pump and the expander when operating with such fluid.

In a study conducted by Qiu G. [20], the main criteria for the proper selection of the working fluid, in decreasing order of relevance, are outlined as the following:

- Not banned by relevant national standards.
- High enthalpy change in the expander; this translates into a higher work output.
- Working fluids should be easy to manage at ambient temperature, where the ambient temperature is not higher than 100 °C.
- Isentropic or “dry” fluids are preferred to avoid superheating in the cycle.
- High latent heat; this makes components in the system smaller.
- Preferential physical properties, such as: chemical stability, high thermal conductivity, low viscosity, and operating at low pressures.
- High safety requirements such as low toxicity, low corrosion, non-flammable, and non-explosive.
- Accessibility and low cost.

**Figure 10:**
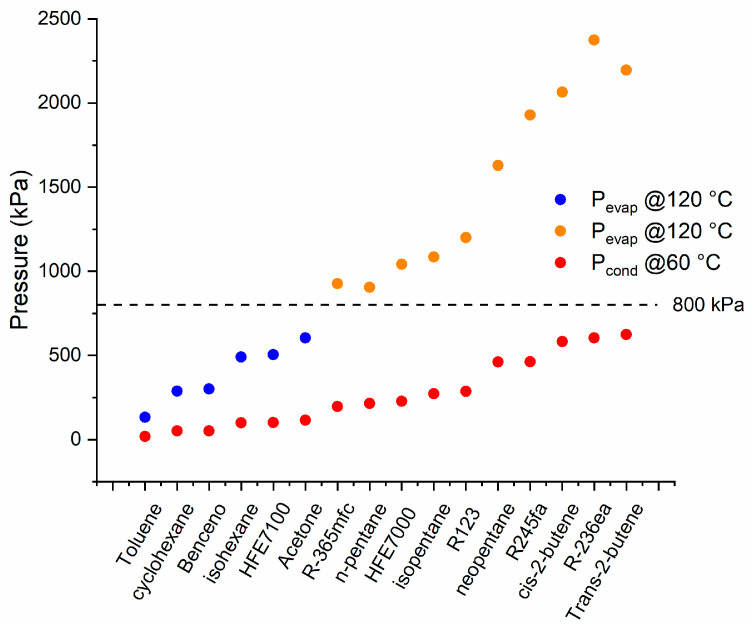
Saturation pressure of working fluids for the ORC conditions.

Through the EES software [35], 29 different organic fluids were analyzed, chosen from Bao et al. and Qui and Rayegan et al. [19,20,21]. It was found that only 16 may be superheated at 120 °C and 800 kPa and subcooled at 60 °C and 100 kPa (Figure 10). Additionally, 13 fluids (not shown) could not be used in the cycle because the saturation pressures obtained at the required temperatures were not feasible in the SDHWS.

#### 5.1.1. Enthalpy Change 

The main characteristic that must be taken into account is a high enthalpy change in the expander, because this means that most of vapor enthalpy may be transformed into useable mechanical work. Therefore, the isentropic enthalpy change in the cycle is evaluated with a set temperature and a pressure not higher than 800 kPa.

**Figure 11:**
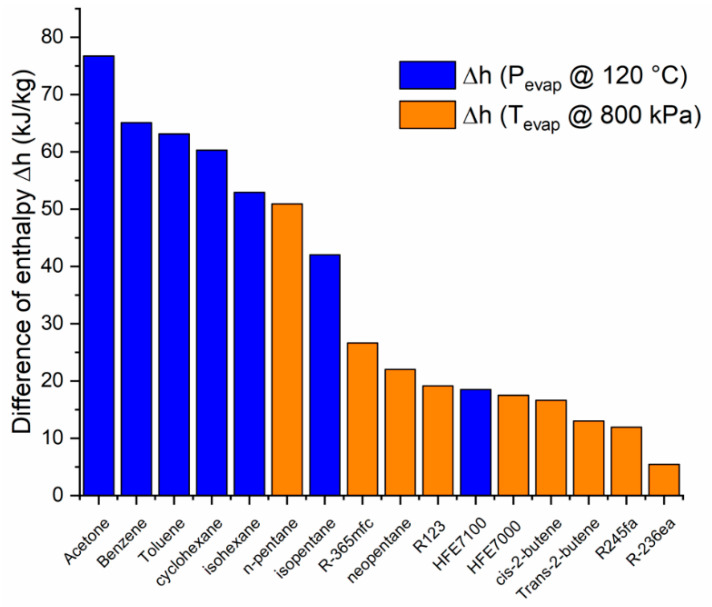
Enthalpy change of working fluids (at a condensation temperature of 60 °C).

**Figure 12:**
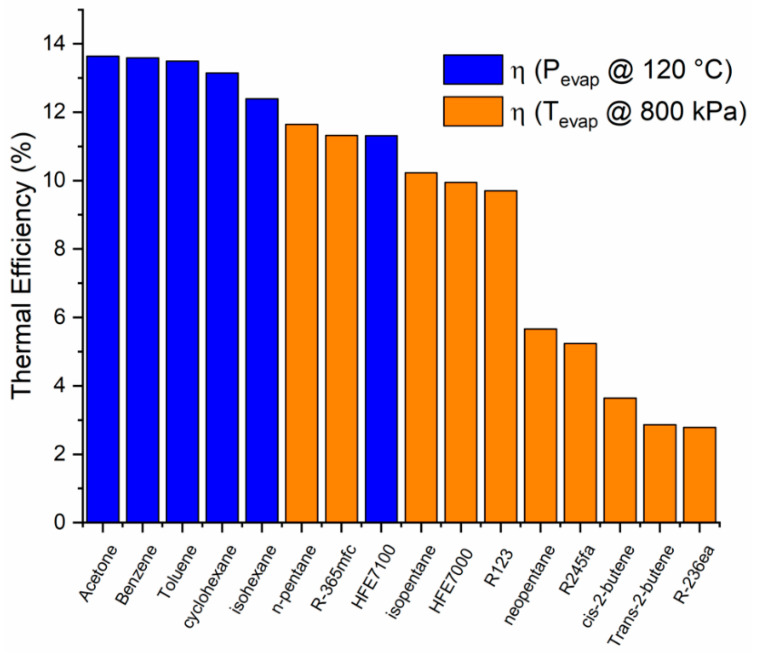
Thermal efficiency of different working fluids (at a condensation temperature of 60 °C).

**Figure 13:**
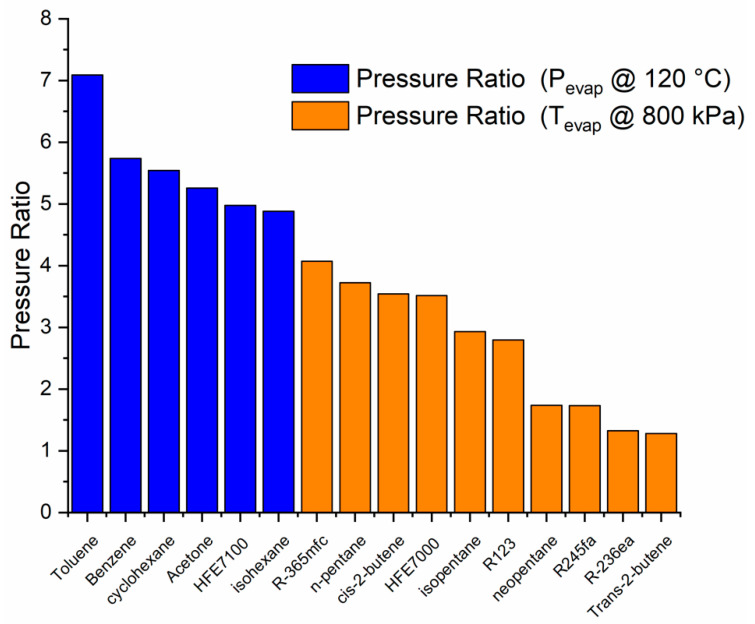
Pressure ratio of different working fluids (at a condensation temperature of 60 °C).

For fluids with a saturation pressure higher than 800 kPa at 120 °C, the enthalpy change is considered limited at this pressure (shown in orange in Figure 10, Figure 11, Figure 12 and Figure 13). A similar consideration is made for cooling down conditions at 60 °C, in order to maintain the operation conditions within the permissible range.

The power produced by the expander is the product of enthalpy change and the mass flow. Therefore, by having a greater enthalpy change, a smaller device (or lower volumetric flow rate) is required.

Figure 11 shows the difference of enthalpy for the different fluids analyzed. It is observed that the five fluids with a higher enthalpy difference between selected temperatures and pressures are: acetone, benzene, toluene, cyclohexane, and isohexane.

#### 5.1.2. Thermal Efficiency

Another important point to consider for proper fluid selection is the thermal efficiency of the ideal cycle; this is the ratio between the enthalpy change at the expander, and the increase of enthalpy at the cycle, as shown in Equation (1):

(1) $$\eta_{ORC,ideal}=\frac{h_{3}-h_{4}}{h_{3}-h_{1}}$$

For a particular saturation temperature difference between the evaporation and condensation states, there will be an enthalpy change. According to Equation (1), the thermal efficiency depends not only on the enthalpy change during the expansion but also on the enthalpy change required for evaporation; that is, the latent heat of vaporization. For each saturation temperature, different working fluids have different latent heats of vaporization due to their intrinsic properties. Therefore, two working fluids can present the same enthalpy change for a given temperature difference but require a different latent heat of vaporization. Under these conditions, the fluid with a bigger latent heat of vaporization will present a lower thermal efficiency.

The thermal efficiencies of the analyzed fluids in Figure 12 show that the five fluids with a higher enthalpy change are those with a higher thermal efficiency. It is important to note that it is not always the fluids with a higher enthalpy change during expansion that will have a higher thermal efficiency. Some examples of this are isopentane, neopentane, and R123, to mention a few.

#### 5.1.3. Expansion Ratio 

For the dimensioning of the expander, it is necessary to know the expansion ratio of the fluid. It may be observed that the fluids with a higher expansion ratio are those with a higher enthalpy change in the cycle, but not in the same order (Figure 13).

It may be observed that there is no direct relationship between the fluid properties and the thermal performance parameters; therefore, it is important to take note of them to make the right choice. From the expansion ratio, it is possible to choose the type of expander that could be used within the cycle. In the next section, the performance of the cycle with the selected working fluid is shown, as well as other parameters required for the selection and suitable design of the expansion device. The selected expander type is a volumetric expansion device, because it is more appropriate for the ORC at small capacities [36] due to its required lower flow rates, higher pressure ratios and rotational speeds, which are lower in comparison to the velocity expander type. The basic expander selection is described in Section 6.

### 5.2. ORC with Acetone as the Working Fluid

At low pressures, the best candidates were hydrocarbons, due to a higher isentropic enthalpy change. Therefore, the pressure in the cycle may be more relevant than the operation temperature at the moment of selecting a working fluid for an ORC.

**Figure 14:**
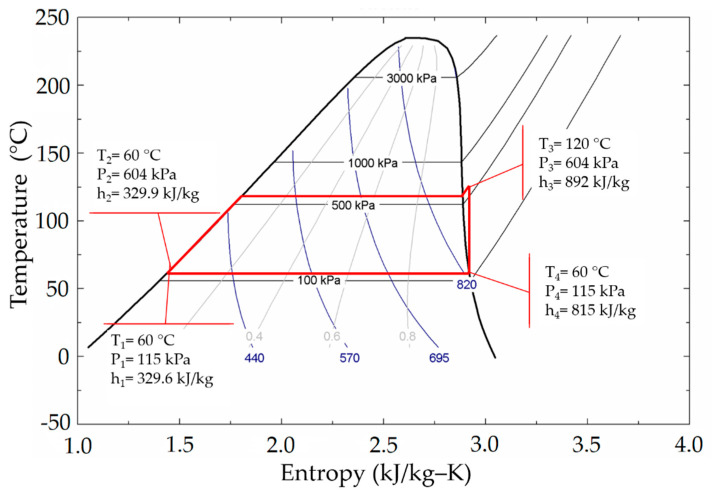
Rankine cycle with acetone as the working fluid.

Acetone was selected as the most suitable fluid to operate in the cycle with the given conditions. In Figure 14, the Rankine cycle is shown, indicating that the saturation pressure is 604 kPa at 120 °C and 115 kPa at 60 °C at the expander output. 

The working fluid is one of the most important factors to take into consideration for the performance of solar collectors [37], because it carries the heat absorbed by the solar collector to the load or to the hot water tank. Therefore, it is important to consider the thermal properties such as thermal conductivity. This property has a major role in the search for equipment with high energy performance [38]. This is because the higher the heat transfer to the fluid, the higher the collector efficiency [39]. Some studies have used acetone as the working fluid in heat pipe collectors, achieving a better performance than with other working fluids [37,40,41,42,43]. These results confirm that acetone is an adequate working fluid to operate with phase changes in solar collectors. 

Among the advantages of using acetone in a SDHWS are it prevents freezing, scaling, fouling and corrosion. This fluid has a low GWP (Global Warming Potential), which is a widely used parameter to show how much heat is trapped in the atmosphere by a greenhouse gas, being in this case 0.5 which is considerably low when compared to typical refrigerants (i.e., R134a GWP = 1300; R410a GWP = 1700). In addition to this, acetone is considered not to have Ozone Depletion Potential (ODP), therefore it is a great candidate for this type of application [44]. The auto-ignition temperature for acetone is 465 °C [45], and since this temperature cannot be reached within the cycle, flammability or explosiveness is avoided. Flammability is also prevented because open flames and sparks are not present in the cycle under typical operating conditions.

Acetone has been experimentally used as a working fluid [1,27,44,46,47]. Other procedures to select working fluids for ORC systems indicate that acetone achieves a high performance for ORC at 130 °C [21].

### 5.3. Analysis of Energy Potential Delivered to the Expander

**Figure 15:**
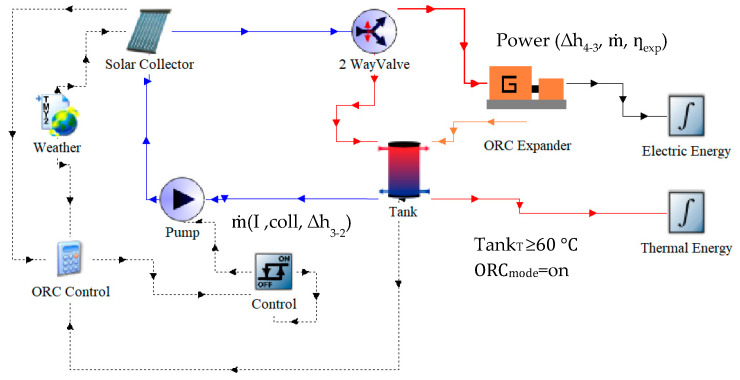
ORC mode scheme in the TRNSYS simulation studio.

To analyze the cogeneration system, the process shown in Figure 15 was carried out, following the methodology described in Section 2. Description of the system, as follows:

Once the hot water tank temperature reaches a preset operation temperature (60 °C) and available irradiance meets CHP energy requirements, the working fluid mass flow that the system can delivered is determined based on: the irradiance, the output thermal power of the collector and the enthalpy required to take the working fluid from subcooled liquid at 60 °C to superheated vapor at 120 °C and 604 kPa at the output of the solar collector.

After the mass flow is calculated, it is possible to obtain the power delivered by the expander by considering the vapor mass flow, as well as the enthalpy drop in the expansion process, and the isentropic efficiency of the expander. Therefore, by the integration over the periods of time (when it is possible to use the cogeneration system), the amount of energy delivered by the expander is calculated.

Once the working fluid has been selected and the heat required to change the state from subcooled liquid at 60 °C to superheated vapor at 120 °C has been defined, the required mass flows are determined through an energy balance. Consequently, it is possible to calculate the volumetric flow that would be obtained under the different operation conditions. With these parameters defined, it is possible to select the most adequate expansion device.

**Figure 16:**
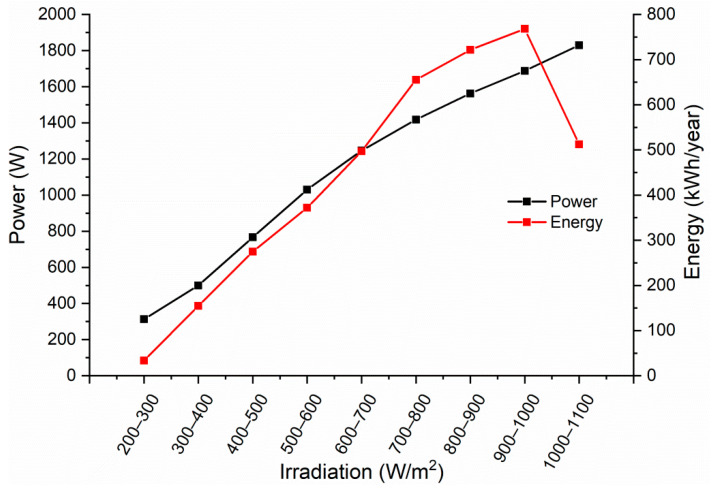
Power and energy given at different irradiance levels during the year.

The collector simulation allows for an estimation of the thermal power, the average useable heat, and the irradiance levels at which the higher energy (2346 kWh/m^2^) and power that can be obtained (Figure 16), in order to optimize the efficiency of the system for these conditions.

In Table 4, the main parameters required for the expander thermodynamic design are listed.

## 6. Expander

Once the ORC configuration (Figure 2) and the system operation conditions (Table 4) have been established and the working fluid has been defined, it is possible to select the best suited expander to work within the cycle. 

The expansion device is one of the four main components of an ORC system, with the most influence over the cycle performance and the total system cost. Adequate selection of the expander depends on the operation conditions, working fluid, size of the system, mass flow, chemical composition of the fluid, temperature difference, and pressure allowed within the system [48]. 

**Figure 17:**
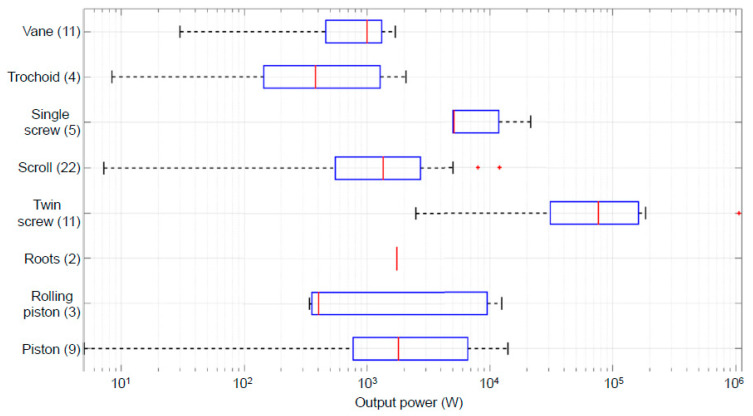
Power range produced by different expansion devices [49].

Figure 17 shows the compilation of different types of expanders that have been used in different power ranges for micro-ORC [49]. The number of studies considered by the authors is shown in parentheses. It may be observed that the better volumetric expansion type for the power considered in this study is the “trochoid” (gerotor) type. One of the advantages of this type of device is that, due to their configuration, there is a low relative speed between the rotor and stator, which generates lower friction losses with respect to other type of positive displacement devices [50].

Currently, there is limited literature that reports on the use of this type of device as an expander for an ORC, however, an ORC system has shown isentropic efficiency of 85% and a global efficiency of 7.7% with a source temperature of 162.2 °C [50]. The expander isentropic efficiency $\eta_{exp}=85\%$ was selected from these results. The maximum capacity of electric power generation of the proposed system is estimated considering the constant efficiency. However, it is important to note that the expansion isentropic efficiency can vary with time and conditions.

In the analysis of the ideal Rankine cycle, the pump and the expander are considered isentropic while the evaporator and condenser do not involve any work. The power of the pump is defined as

(2) $$W_{pump,in}=\dot{m}\left(h_{2}-h_{1}\right)$$

And the power of the expander is

(3) $$W_{exp,out}=\dot{m}\left(h_{3}-h_{4}\right)$$

However, one of the major deviations within the ideal Rankine cycle is the irreversibilities occurring within the pump and the expander. To obtain more accurate results, it is necessary to take into consideration the work involved within the cycle, and also the isentropic efficiencies.

For this particular study a pump efficiency of $\eta_{pump}=65\%$ [12,15,16,51,52] is considered. The power required by the pump can be calculated by

(4) $$W_{pump}=\frac{\Delta h_{2-1}\cdot \dot{m}}{\eta_{pump}}$$

The expander would be considered to achieve 85%, so the power of the expander can be calculated by

(5) $$W_{\exp}=\Delta h_{4-3}\cdot \dot{m}\cdot \eta_{exp}$$

When considering Equations (4) and (5), it is possible to calculate the amount of power generated by the expander, as well as the power required by the pump to maintain the flow of the working fluid in the cycle. To obtain the maximum power of the expander the maximum flow from Figure 16 is considered in Equation (5), which gives a value of 245 W.

The efficiency of the ORC system can be calculated by considering these isentropic efficiencies, the constant temperatures and pressures in the cycle, obtaining 84.4% of the ideal ORC, as given below:

(6) $$\eta_{ORC}=\frac{W_{exp}-W_{pump}}{\dot{m}\left(h_{4}-h_{3}\right)}$$

The efficiency from solar to electric power can be obtained as follows:

(7) $$\eta_{solar}=\frac{E_{elect}}{{\dot{Q}}_{u}}$$

The CHP efficiency is given by

(8) $$\eta_{CHP}=\frac{{\dot{W}}_{net}+{\dot{W}}_{heat}}{{\dot{Q}}_{u}}$$

The proposed system has 2346 kWh/m^2^ annual irradiation, and a 2.84 m^2^ collector surface area; it is possible to generate a useful electric energy of 443 kWh, with a global efficiency of solar to electric power of 6.65% (Equation (7)). Additionally, the overall efficiency of the cogeneration system is 56.35% (Equation (8)) when considering CHP generation.

## 7. Conclusions

In this paper the application of an ORC to a SDHWS was presented and analyzed with the purpose of observing the cycle feasibility, the system energy balances, and the proper working fluid selection.

The collector performance was analyzed when operating at different temperatures, and it was observed that as the operation temperature increases, the collector efficiency decreases, but the theoretical efficiency of the ORC increases. Thus, a point of operation was determined to meet the needs of both systems.

Different working fluids were analyzed below the maximum collector permissible pressures, showing that hydrocarbons (HCs) provide a better performance than other organic fluids at low pressures (100–1000 kPa). A multiple criteria analysis was conducted (i.e., enthalpy change, thermal efficiency, and expansion ratio), showing a similar performance, with some variations. This study found that acetone is the most suitable working fluid because of its higher enthalpy change under the operating conditions.

This design method allows evaluation of the useable heat generated by a solar system under different operation conditions, selecting the most suitable working fluids for the cycle and therefore selecting the best design conditions for an ORC cycle expander.

It is technically feasible to implement a CHP system with the proposed configuration with a better utilization of the heat that went unutilized by the SDHWS in a second application through a low temperature ORC. With a solar collector area of 2.84 m^2^ it is possible to generate 443 kWh of electric power annually with a solar to electric efficiency of 6.65%, or an overall cogeneration system efficiency of 56.35%. 

The objective of this study was to analyze the technical feasibility of the proposed cogeneration system, in order to obtain the main parameters for the design of the expander that can convert part of the heat delivered by the SDHWS into mechanical energy.

Future work will include the development of said expander, as well as the implementation of the ORC system proposed within the SDHWS in order to validate the proposed technique. Once validated, larger capacity systems can be designed and developed.

## 8. Patents

Patents related to this work:

- Cogeneration System for Integration into Solar Water Heating Systems, U.S. Patent, Application Number: 16,208,666.
- Dispositivo de cogeneración para integración en sistemas de calentadores solares de agua, IMPI: MX/E/2017/094793.

## Figures and Tables

**Table 1:** Performance of different Organic Rankine Cycles (ORCs).

Author	Output Power	Isentropic Efficiency (%)	Global Efficiency (%)
Helvaci and Khan 2016 [12]	135.96 W	58.66	3.81
Taccani et al. 2016 [13]	670 We	62	8
Vittorini et al. 2018 [14]	700 W	Not shown	3 *
J. Freeman et al. 2017 [15]	~1 kWe	75 *	7.3
Cioccolanti, et al. 2017 [16]	2 kWe	60 *	4.98

* assumed by the author.

**Table 2:** Basic specifications of the system components.

**Solar Collector**		**Cooling Coil**	
Aperture area	2.84 m^2^	Length	18.6 m
Fluid capacity	0.790 L	Area	0.929 m^2^
Flow rate	0.0333 L/s	Coil material	cooper
Max flow rate	0.25 L/s	Coil internal diameter	1.27 cm
Max operating pressure	800 kPa	Coil external diameter	1.59 cm
**Hot Water Tank**		**Expander**	
Capacity	270 L	Max. Output Power	245W
Height	1.5 m	Nominal expander power	194 W
Internal diameter	0.45 m	Nominal Volumetric flow	850 L/h
Insulation thickness	0.047 m	Expansion ratio	4.85
Insulation conductivity	0.036 W/mK	Isentropic efficiency	0.85
**Pump**		**Design Point of the ORC**	
Required power for ORC	1.5 W	Irradiance	950 W/m^2^
Flow rate	15.4 L/h	Collector thermal efficiency	62.50%
Input pressure	115 kPa	Output collector power	1687 W
Output pressure	604 kPa	Expander output	194 W
Fluid temperature	60 °C	Global efficiency	7.20%

**Table 3:** Typical residential hot water consumption [29].

Usage	Amount of Hot Water (L)	Daily Total Consumption per Household (L)
1 x Handwashing	3	(12 Uses) 36
1 x Shower	35	(4 Uses) 140
1 x Dishwashing	20	(2 Uses) 40
Cleaning	3 per person per day	12
Cooking	2 per person per day	8
Total Daily Requirement: 236 L		

**Table 4:** Main parameters for the expander design.

**Working Fluid: Acetone**			
	Inlet	Outlet	Difference
Pressure	604 kPa	115 kPa	P_1_/P_2_ = 5.25
Temperature	120 °C	60 °C	∆T = 60 °C
Enthalpy	892 kJ/kg	815 kJ/kg	∆h = 77 kJ/kg
Viscosity	10.18 × 10^−6^ Pa∙s	23.12 × 10^−5^ Pa∙s	∆μ = 22 × 10^−5^ Pa∙s
Density	12.68 kg/m^3^	2.612 kg/m^3^	ρ_1_/ρ_2_ = 4.85
**Operation Range**			
Volumetric Flow	V̇_min_ =180 L/h	V̇_max_ = 900 L/h	V̇_nominal_ = 850 L/h
Inlet Heat	Q_min_ = 310 W	Q_max_ = 1830 W	Q_nominal_ = 1690 W

## Nomenclature

*E*	total energy
*h*	specific enthalpy (kJ/kg)
*I*	solar radiation (W/m^2^)
$\dot{m}$	mass flow rate (kg/s)
*P*	pressure (kPa)
*Q*	heat (W)
${\dot{Q}}_{u}$	useful heat gain (W)
*s*	entropy (kJ/kg)
*t*	time (s)
*T*	temperature (°C)
*U*	overall heat transfer coefficient (W/m^2^K)
$\dot{V}$	volumetric flow rate (m^3^/s)
*W*	work (W)
**Subscripts**	
*CHP*	combined heat and power
*coll*	solar collector
*cond*	condensation
*elect*	electric
*evap*	evaporation
*exp*	expander
*ORC*	organic Rankine cycle
*SDHWS*	solar domestic hot water system
**Greek Symbols**	
∆	difference
*Ƞ*	efficiency
*μ*	dynamic viscosity
*ρ*	density

## References

[1] Kim W.T., Song K.S., Lee Y. (1998). Design of a two-phase loop thermosyphon for telecommunications system (I). KSME Int. J..

[2] Fokaides P.A., Christoforou E.A., Kalogirou S.A. (2014). Legislation driven scenarios based on recent construction advancements towards the achievement of nearly zero energy dwellings in the southern European country of Cyprus. Energy.

[3] Nizetic S., Coko D., Marasovic I. (2014). Experimental study on a hybrid energy system with small- and medium-scale applications for mild climates. Energy.

[4] Kougias I., Szabó S., Nikitas A., Theodossiou N. (2019). Sustainable energy modelling of non-interconnected Mediterranean islands. Renew. Energy.

[5] Martínez-Hervás M., Sendra J.J., Suárez R. (2017). Towards an energy assessment on an urban scale for retrofitting the housing stock in Mediterranean cities. Procedia Environ. Sci..

[6] Tsalikis G., Martinopoulos G. (2015). Solar energy systems potential for nearly net zero energy residential buildings. Sol. Energy.

[7] Bracco R., Clemente S., Micheli D., Reini M. (2013). Experimental tests and modelization of a domestic-scale organic rankine cycle. Energy.

[8] Sahar S., Fereshteh A. (2014). Energy and exergy assessments of modified Organic Rankine Cycles (ORCs). Energy Rep..

[9] Bianchi M., De Pascale A. (2011). Bottoming cycles for electric energy generation: Parametric investigation of available and innovative solutions for the exploitation of low and medium temperature heat sources. Appl. Energy.

[10] Quoilin S., Van Den Broek M., Declaye S., Dewallef P., Lemort V. (2013). Techno-economic survey of Organic Rankine Cycle (ORC) systems. Renew. Sustain. Energy Rev..

[11] Dickes R., Ziviani D., de Paepe M., van den Broek M., Quoilin S., Lemort V. ORCmKit: An open-source library for organic Rankine cycle modelling and analysis. Proceedings of the 29th International Conference on Efficiency, Cost, Optimization, Simulation and Environmental Impact of Energy Systems.

[12] Helvaci H.U., Khan Z.A. (2016). Experimental study of thermodynamic assessment of a small scale solar thermal system. Energy Convers. Manag..

[13] Taccani R., Obi J.B., De Lucia M., Micheli D., Toniato G. (2016). Development and experimental characterization of a small scale solar powered Organic Rankine Cycle (ORC). Energy Procedia.

[14] Vittorini D., Antonini A., Cipollone R., Carapellucci R., Villante C. (2018). Solar thermal-based ORC power Plant for micro cogeneration—Performance analysis and control strategy. Energy Procedia.

[15] Freeman J., Guarracino I., Kalogirou S.A., Markides C.N. (2017). A small-scale solar organic Rankine cycle combined heat and power system with integrated thermal-energy storage. Appl. Therm. Eng..

[16] Cioccolanti L., Tascioni R., Arteconi A. (2017). Simulation analysis of an innovative micro-solar 2kWe Organic Rankine Cycle plant for residential applications. Energy Procedia.

[17] Frutiger J., Andreasen J., Liu W., Spliethoff H., Haglind F., Abildskov J., Sin G. (2016). Working fluid selection for organic Rankine cycles—Impact of uncertainty of fluid properties. Energy.

[18] Schilling J., Tillmanns D., Lampe M., Hopp M., Gross J., Bardow A. (2017). Integrated thermo-economic design of ORC process, working fluid and equipment using PC-SAFT. Comput. Aided Chem. Eng..

[19] Bao J., Zhao L. (2013). A review of working fl uid and expander selections for organic Rankine cycle. Renew. Sustain. Energy Rev..

[20] Qiu G. (2012). Selection of working fluids for micro-CHP systems with ORC. Renew Energy.

[21] Rayegan R., Tao Y.X. (2011). A procedure to select working fluids for Solar Organic Rankine Cycles (ORCs). Renew. Energy.

[22] Chavez D.A.L., Chacon R.B. (2019). Cogeneration System for Integration Into Solar Water Heating Systems. U.S. Patent.

[23] Apricus (2016). ETC Solar Collector Product Overview.

[24] Farrington R.B., Bingham C.E. (1986). Testing and Analysis of Immersed Heat Exchangers.

[25] INEGI Encuesta INTERCENSAL 2015. https://www.inegi.org.mx/contenidos/programas/intercensal/2015/doc/eic_2015_presentacion.

[26] ASPE Domestic Hot Water Systems 2015.

[27] Kalogirou S.A. (2013). Solar Energy Engineering: Processes and Systems.

[28] TÜV Rheinland (Shanghai) Co., Ltd. (2014). Qualification of a Solar Collector in Accordance with DIN EN 12975-1: 2011; DIN EN 12975-2: 2006.

[29] Cruickshank C.A., Harrison S.J. (2010). Heat loss characteristics for a typical solar domestic hot water storage. Energy Build..

[30] ASHRAE (2011). 2011 ASHRAE HANDBOOK HVAC Applications.

[31] Marini D., Buswell R., Hopfe C. Estimating waste heat from domestic hot water systems in UK dwellings. Proceedings of the International Building Performance Simulation Association.

[32] Xu L.C., Liu Z.H., Li S.F., Shao Z.X., Xia N. (2019). Performance of solar mid-temperature evacuated tube collector for steam generation. Sol. Energy.

[33] Sabiha M.A., Saidur R., Mekhilef S., Mahian O. (2015). Progress and latest developments of evacuated tube solar collectors. Renew. Sustain. Energy Rev..

[34] Madduri A., Loeder D., Beutler N., He M., Sanders S. Concentrated evacuated tubes for solar-thermal energy generation using stirling engine. Proceedings of the 2012 IEEE Energytech.

[35] EES: Engineering Equation Solver F-Chart Software: Engineering Software. http://www.fchart.com/ees/.

[36] Imran M., Usman M., Park B.S., Lee D.H. (2016). Volumetric expanders for low grade heat and waste heat recovery applications. Renew. Sustain. Energy Rev..

[37] Shafieian A., Khiadani M., Nosrati A. (2019). Strategies to improve the thermal performance of heat pipe solar collectors in solar systems: A review. Energy Convers. Manag..

[38] Pinto R.V., Fiorelli F.A.S. (2016). Review of the mechanisms responsible for heat transfer enhancement using nanofluids. Appl. Therm. Eng..

[39] Farhana K., Kadirgama K., Rahman M.M., Ramasamy D., Noor M.M., Najafi G., Samykano M., Mahamude A.S.F. (2019). Improvement in the performance of solar collectors with nanofluids—A state-of-the-art review. Nano Struct. Nano Objects.

[40] Eidan A.A., AlSahlani A., Ahmed A.Q., Al-fahham M., Jalil J.M. (2018). Improving the performance of heat pipe-evacuated tube solar collector experimentally by using Al2O3 and CuO/acetone nanofluids. Sol. Energy.

[41] Föste S., Schiebler B., Giovannetti F., Rockendorf G., Jack S. (2016). Butane heat pipes for stagnation temperature reduction of solar thermal collectors. Energy Procedia.

[42] Shafieian A., Khiadani M., Nosrati A. (2018). A review of latest developments, progress, and applications of heat pipe solar collectors. Renew. Sustain. Energy Rev..

[43] Ersöz M.A. (2016). Effects of different working fluid use on the energy and exergy performance for evacuated tube solar collector with thermosyphon heat pipe. Renew. Energy.

[44] Ordaz-Flores A., García-Valladares O., Gómez V.H. (2012). Findings to improve the performance of a two-phase flat plate solar system, using acetone and methanol as working fluids. Sol. Energy.

[45] Product S., Information C., Kit G. Safety Data Sheet Acetone. **2015**, *77*, 1–4. https://www.labchem.com/tools/msds/msds/LC10420.pdf.

[46] Islam M.A., Khan M.A.R., Sarkar M.A.R. (2005). Performance of a Two-Phase Solar Collector in Water Heating. J. Energy Environ..

[47] Szymański P., Mikielewicz D. (2017). Experimental study of pressure rise at the evaporator of capillary pumped loop with acetone and water as working fluids. Exp. Therm. Fluid. Sci..

[48] Johnson I., Choate W.T., Davidson A. (2008). Waste Heat Recovery: Technology and Opportunities in U.S. Industry.

[49] Lemort V., Legros A. (2017). Positive displacement expanders for Organic Rankine Cycle systems. Organic Rankine Cycle (ORC) Power Systems.

[50] Mathias J.A., Johnston J.R., Cao J., Priedeman D.K., Christensen R.N. (2009). Experimental testing of gerotor and scroll expanders used in, and energetic and exergetic modeling of, an Organic Rankine Cycle. J. Energy Resour. Technol..

[51] Xia X.X., Wang Z.Q., Hu Y.H., Zhou N.J. (2018). A novel comprehensive evaluation methodology of organic Rankine cycle for parameters design and working fluid selection. Appl. Therm. Eng..

[52] White M.T., Oyewunmi O.A., Chatzopoulou M.A., Pantaleo A.M., Haslam A.J., Markides C.N. (2018). Computer-aided working-fluid design, thermodynamic optimisation and thermoeconomic assessment of ORC systems for waste-heat recovery. Energy.

